# Animal Welfare and the Acknowledgment of Cultural Differences

**DOI:** 10.3390/ani12040474

**Published:** 2022-02-15

**Authors:** Arlene Garcia, John J. McGlone

**Affiliations:** 1 School of Veterinary Medicine, Texas Tech University, Amarillo, TX 79106, USA; 2 Laboratory of Animal Behavior, Physiology and Welfare, Animal and Food Sciences Department, Lubbock, TX 79409, USA; john.mcglone@ttu.edu

**Keywords:** pigs, welfare, sustainability

## Abstract

**Simple Summary:**

Most pigs worldwide are in modern, commercial, indoor farms. There is debate on whether or not modern pig production systems meet all the needs of the animals. Pigs are able to perceive and feel and have mental capabilities that warrant attention to their welfare. The degree of on-farm animal welfare oversight varies around the world. Science is used to set animal welfare rules. However, science sometimes conflicts with cultural values (ex., religious slaughter). The focus of many animal welfare rules do not address the most significant problems on modern commercial pig farms. We believe that we should use science to determine the animal welfare needs of farm pigs but with a healthy respect for cultural differences in the ethics of animal care. We should also prevent economic neo-colonialism from forcing Western views on other cultures.

**Abstract:**

Pigs are considered sentient beings that have a mental capability that warrants attention to their welfare. Cultural values towards animal welfare differ in world regions. Still, authors have argued for worldwide harmonization of animal welfare rules. At the same time, the focus of many animal welfare rules do not address the most significant problems on modern commercial pig farms. The foci of animal welfare rules are often on space (quantity and quality), acute painful practices, equipment, and caretaker behavior. However, most serious animal welfare issues are related to episodic events such as compromised pigs, lack of appropriately skilled staff, and human behavior (or lack thereof) towards animals. Modern technologies such as image, sound, and building oversight by automated systems can potentially provide better individual pig care. The future should bring us solutions to identify and resolve episodic negative animal welfare events. The other issues of space and painful practices are best improved by using science-based solutions. We propose that science be the key resource to making animal welfare decisions, but with a healthy appreciation and respect for cultural differences in our views of animals and the economic impact of rules. Colonialism is not viewed positively today, and economic neo-colonialism should not be allowed to replace it. Respect for cultural differences should play a role in animal welfare rules within and among countries.

## 1. Introduction

In 2019, the value of U.S. pork and pork product exports to the world reached a record $7.0 billion, up 9% from the previous year [1]. Up until recently, pork was far-and-away the meat consumed in the largest quantity in the world. Chicken consumption is increasing while disease challenges suppressed pig numbers in recent years.

Pork consumption took a worldwide hit in 2018 when Asia (especially China) was exposed to African Swine Fever (ASF). Asia lost on the order of half of their pigs in a short period. This loss of product caused a worldwide shortage of pork. Naturally, people consumed other meats. Chicken consumption increased worldwide and it is expected to overtake pork as the most-consumed meat in the world. However, with nearly 1 billion pigs produced in the world each year, the swine industry is large, complex, efficient, and a major part of the culture of many people.

Western-based cultures, including North and South America, have developed production systems based on the constraints of their geography and culture. However, today, pork production is rapidly growing in Asia and to some extent Latin America, more so than in North America and Europe. Innovation in production technology comes mostly as new farms are built.

By Western cultures (itself a biased term), we mean predominantly European views that are held by people from Europe and North and South America. We acknowledge that views of animals vary even within European countries—the views, for example, of people from Italy and Sweden can be quite different about animals [2]. Even the current animal views of people from Spain and Mexico, while similar in some ways, are very different in other ways [3].

We often confuse geography with culture. Sure, more Muslims live in the Middle East than other religions, but not just Muslims live there, and Muslims are found all around the world. We are not attempting to discuss religious beliefs or politics; however, certain religions are more common in some geographies and so the overlap is unavoidable. Here, we attempt to focus on cultural differences, not politics or religion.

A newer term is cultural competency [4]. Some authors believe that if one does not agree with an animal welfare practice (like spaying dogs), then they are not culturally competent [4]. The logic behind these thoughts are that the more education you provide, the more your audience holds your views, the more culturally competent the audience would be.

Cultural differences in views of animal welfare are known to be quite different around the world—some differences are large and some small. Although, not enough time and effort has gone into describing large cultural differences in views of animals. As an example, to demonstrate the difference between cultures, we did a survey of local workers on a large commercial pig farm [5] to determine if origin (Hispanic vs. Caucasian genetics and upbringing; one cannot easily separate nation of origin from cultural/socio-economic development) impacted whether pet dogs were kept indoors or outdoors. We found that workers of Hispanic descent were more likely to house pet dogs outdoors, while non-Hispanic white people more often kept pet dogs indoors. This is a small example of cultural differences of views towards animals.

While not well studied, the “Western” view is that we should use our economic muscle to impose our animal welfare “ethics” on people in countries with developing economies [6]. We do not believe the intent was to invoke colonialism tactics, but that is the effect—using economics to force other cultures to adopt our rules, even if such rules are foreign to their way of thinking. While some authors have proposed this view [6], other question if this is the best approach [7].

## 2. Animal Welfare Perspectives in the World

Most writings and experimentation about animal welfare are produced by authors from cultures of European or Western ancestry. In the 1960s, Ruth Harrison’s book Animal Machines brought the issue of animal welfare in intensive farming, including pigs to the public [8]. This book caused the UK government to get involved in farm animal welfare. The ideas spread among other peoples of European descent, including North and South America. The two ways reported to approach animal welfare are: (1) how the animal feels and (2) how the animal performs—are both approaches based on Western thinking. However, what about other cultural views of animal welfare? The diversity of attitudes towards animals is influenced by different cultures with key drivers that include religion, general education level, economic status, and use of animals, as determined by the climatic and historical situation of a region [9]. Most people think that extremes of poor welfare in animals are unacceptable and people should strive for good animal welfare [10]. This way of thinking has led to animal welfare science. Animal welfare science and animal welfare scientists have been defined, respectively, as scientific investigations of animals and the people who conduct such investigations, but not to the broader discipline of animal welfare, which includes ethics and ethicists [11,12].

People who believe animals have rights are not uniform in their views. Some may think animals have the right to life (not to be killed and eaten), while others may believe animals do not have the right to life, but they do have the right to not suffer. Regardless of how many and which rights one ascribes to animals, the animals have a certain level of welfare in each situation. To improve animal welfare is not dependent on ascribing rights to animals. Animal welfare scientists can study animal behavior and physiology to determine if one or another practice is better for the animal. They have no need to first say the animal has any rights. However, after one has data to show that one practice is better than another, one must have a business, moral, ethical, or legal construct to then require that the worse practice is not used.

Our view, which is not common among animal welfare scientists, is that people from countries with developing economies should have an equal voice in determining how animals are treated. A certain human society should not be forced to apply animal welfare standards that they do not believe in, are unimportant in their view, or are unable to adopt. To force people to adhere to Western animal ethics is a way of destroying other cultures. The world is currently dealing with this issue. Here we use as an example the issue of humane slaughter. In this example, Western and Middle Eastern cultural/religious beliefs are quite different.

In the USA and the EU, laws like the USA Humane Slaughter Act requires that livestock be rendered instantaneously insensible before being exsanguinated, except in the case of religious slaughter. To be made instantaneously insensible seems logical and better for the animals. Rules of Kosher and Halal slaughter support the view that animals should not be stunned prior to exsanguination. In an oversimplification of this religious/cultural view, they believe animals should experience death and should have blood removed from the carcass by a qualified person with a very sharp, long knife [13]. To force cultures to adopt what is called humane slaughter goes against the religious and cultural views of millions of people particularly of Jewish and Muslim faiths; however, this is not just a religious issue in that some people in the Middle East may not practice a religion, but they follow the culinary customs of their culture. Some countries have forced ritual slaughter to be abandoned, while other efforts have looked for a compromise, such as using a level of restraint that does not involve shackling and hoisting a live animal [14]. New Zealand banned ritual/religious slaughter in 2010. This caused an outcry from the Jewish community, claiming that this law was a threat to their culture [13].

In 2022, we have strong, yet contrasting views of multi-culturalism vs nationalism. When countries (or even regions) seek autonomy, they seek the protection of their culture and nationalism becomes the battle cry. Being forced to attend to society issues like climate change or animal welfare causes some nations to pull away from practices that may cause economic, cultural, or social harm. Cultures vary widely on their views towards animals (though this needs more study). Cultural differences can be found among continents, countries, and regions.

In the last 50 years, farm animal welfare discussions and laws have spread around the world. Yet, the approach to regulating animal welfare varies around the world today. Some countries have strict animal welfare laws and regulations, while many other countries have very few of them. The USA has avoided animal welfare national laws in part because the marketplace has its own rules (like food retailers) that are viewed as sufficient.

An alternative view is that Western nations require that countries with developing economies or non-Western nations develop their own animal welfare standards without influence from Western cultures—even this may be culturally offensive. The risk is that some nations will not consider animal welfare to be very important. Is it our role to convince them to think it is important or is it better for them to have their own cultural human–animal views that are consistent with their culture?

### Animal Welfare Perspectives

The loudest voices about animal welfare, both in mass and volume, come from Western cultures and nations, especially Northern Europe and North America. Multiple Western concepts are proposed for how to measure and manage animal welfare, but the result is a bewildering array of laws, regulations, guidelines, and audits that vary from country to country, and even vary within a country. The rules in Germany are not the same as in Greece and certainly not the same as in the U.S., Mexico, or Brazil. The West cannot decide, after four decades of science and thinking, how to uniformly define, measure, and manage pig welfare. Still, writers and non-governmental organizations (NGOs) argue we should use the West’s economic power to cause other nations to adopt Western views by economic pressures [6]. Instead, we suggest to use science to determine the animal welfare needs of pigs, including a healthy respect for cultural differences in the ethics of animal care.

The swine industry is experiencing massive growth in pig numbers in Brazil, China, and parts of South East (SE) Asia. Like many countries, economic drive is a major contributor to how animals are raised. Furthermore, concepts like how a pig feels [15] or if it is bored [16] are not highly considered in these non-Western cultures, and these are certainly not concepts that enter into the design of new buildings being developed. We are not aware of publications from non-Western writers offering alternative views on these concepts; often it may be because they are not published or their work is not published in English [17]. Yet, while we worry about what non-Westerners are doing or not doing, Western writers many times cannot agree on animal welfare concepts, such as how to measure and manage how pigs feel [15], as some consider how they perform [18] is more telling about the pigs’ overall experience, rather than how they feel.

As pig farms are built at a rapid rate around the world, the incorporation of technologies such as precision livestock methods, enhanced environmental protection, and heightened biosecurity should improve pig welfare.

## 3. Pig Welfare Issues

### 3.1. Wilful Acts of Abuse

Wilful acts of abuse are described in detail in most well-known animal welfare audit standards, such as the North American Meat Institute [19], the Common Swine Industry Audit [20], and other standards. Wilful acts of abuse on-farm are human behaviors that are outside of normal production practices and that are unacceptable and unjustified. These typically include hitting/beating, dragging (except in cases of immediate danger), throwing of animals, excessive prod use or use on sensitive parts of the animals (such as eyes, ears, nose, genitals, and rectum), driving animals off high ledges, and animal neglect (such as intentional failure to provide water and food). Some animal welfare auditing standards go beyond observing for these wilful acts of abuse. These additional observations include things such as failure to provide timely euthanasia [20], and can be site-specific, such as farm vs meat plant standards. Both types of standards use the same general concepts to ensure animals are not suffering. Yet, some of these standards are not science-based but it is intuitive (and correct) that hurting an animal and not tending to an animal in need is wrong, no matter what the science might say. Using intuition is better than not using anything to make decisions; however, intuition is fraught with error and bias, including our strong desire to anthropomorphize about animals. Scientific evidence is a far better way to make decisions, unless cultural differences trump the science. Must we require scientific evidence to conclude that beating an animal is bad for its welfare? Usually not; common sense plays an important role in animal care.

### 3.2. Animal Welfare Auditing

For the past 20 years, buyers in the USA have been requiring that their suppliers undergo animal welfare inspections and audits [21]. Animal welfare auditing has led to many improvements, such as reduction in electric prod use, humane handling, stunning, facility management (better lighting, flooring, and facility and equipment maintenance), and employee training, among other improvements [21]. The use of animal-based measures (such as tail biting, prolapses, shoulder sores, and other injuries and problems) during audits also help identify areas of concern, which may require improvement to maintain optimum animal health and welfare. Animal welfare auditing improves animal welfare and provides a repertoire of benefits, including economic benefit to the swine industry, as audits add value to pork products.

### 3.3. Compromised Pigs—The Largest Most Direct Animal Welfare Issue on the Farm

When an animal becomes sick or injured and is at risk of experiencing pain and suffering leading to less-than-optimal welfare, it is known as “compromised”. Proper and timely identification of these animals is critical to prevent poor welfare. Long-term chronic conditions can severely compromise animal welfare. Some of these conditions include but are not limited to, high numbers of lame pigs [22,23], which can have negative effects on handling and transport [24]; lung lesions [25]; and problems with caring for excess piglets from highly prolific sows [26]. These conditions can have detrimental effects on animal health and welfare, animal longevity, and employee workload, which can all affect the economics of the swine industry.

Science is not yet ready to compare the welfare of a sow in a gestation crate with a compromised sow. The compromised sow has an acute health problem that involves immediate pain and/or discomfort. If 1000 sows are bored in a crate, does this negative welfare compare with a sow with an untreated broken leg? The compromised sow requires immediate action on the part of its human caregiver. We can argue about the welfare of the sow in a crate, but there is no argument about the compromised sow.

In general, if the prognosis is poor, the animal meets certain euthanasia criteria, or the value of an individual animal is low, then immediate euthanasia should be the preferred approach instead of prolonged isolation and treatment [27]. However, identification of these animals comes with experience (this issue may improve over time with technological advances). Employees must be trained to recognize multiple health disorders, aetiologies, and know what treatments to use. The ideal situation would be for a professional (such as a veterinarian or a manager that has been previously trained by a veterinarian) to train workers on how to identify each specific health condition and then employees should follow the designated protocol, which was developed by the farm veterinarian for appropriate medical treatment of the animal or proceed with humane euthanasia. Unfortunately, as farms get larger and the lack of agricultural employees grows, these compromised animals are less likely to be identified, treated, or euthanized in a timely manner and thus, they suffer. The development of validated pain scales for pigs has been slow. In addition, many scales require simplification for field application [28]. Again, individuals must be trained to use these scales and know the proper protocols to follow once the pig is identified as compromised. One of the current issues in the U.S. is that more than half of the agricultural workforce is Hispanic [29] and many of these workers do not speak English. It is important to conduct training in the target language for the workforce to understand how to identify and manage compromised pigs. Otherwise, their actions are based on learned behaviors from other employees. Every individual must be trained in their native language to ensure they are abiding by their company’s protocols.

### 3.4. Sow Housing

Gestating sows are housed in two types of systems, either individual gestation stalls or group housing. The gestation crate has been banned in the EU and in several USA states due to animal welfare concerns. The few new farms that are being built in Europe today do not use gestation crates, yet they remain fairly common in North America, in compliance with national legislation. New farms being built in Asia use gestation crates almost exclusively. The rapid growth of new farms in Asia will move the percentage of sows in the world in crates higher. As time moves on, the world will have more sows in gestation crates than we have now, in spite of efforts by some governments, activists, and businesspeople to move away from the gestation crate. This trajectory could change, however, as Asian countries adopt animal welfare rules similar to those adopted by the EU. South Korea recently banned gestation crates starting six weeks after breeding (by the year 2030). Korean scientists replicated USA and EU studies that show, essentially, that the performance of sows is equivalent in crates vs group housing [30] just as was reported earlier [31]. Performance is important to consider when sows are placed in group housing compared to gestation stalls because post-mixing aggression and feeder-related aggression [32] can lead to reduced feed intake, adversely affect reproductive performance [33], cause injuries, and stress, as increased levels of cortisol concentrations have been reported [34]. Based on the body of scientific knowledge, one cannot find large differences in the performance of sows in crates vs group pens (yet, large behavioral differences among sows in the two systems are quite different). When pig performance is equal between two systems (crate vs group pen), one can reach one of two conclusions. First, the welfare of the sow in the gestation crate is not different from those in group pens; therefore, either system is fine (this is the current view of most USA pig farmers). Alternatively, if sow welfare in crates or group pens are not different, why not use the system that some consumers prefer and that is seen as more animal welfare friendly (such as group housing)? Regardless of the system, one must consider the behavioral needs of the pigs for optimal welfare. Can we accommodate these needs with either system?

### 3.5. Indoor vs. Outdoor

Many of us like to see animals outdoors on green grass in a pleasant setting. To see a sow grazing on a green field and a sow in a gestation stall brings up emotions in some people. They might not even be able to say why they do not like the stall. No amount of science would convince a given emotionally engaged person that the sow is as well off in the crate as in the green field. Moreover, comparing the welfare of a sow in a field vs a crate is a challenge. For example, people (and many scientists) do not like that crated sows show stereotyped oral-nasal-facial behaviors (such as bar biting). Objectively, bar biting is an oral-nasal-facial behavior that seems to serve no obvious purpose. However, when one learns that sows in pastures spend a large amount of time engaged in oral-nasal-facial behaviors manipulating non-digestible materials [35]—even more than when they are in a crate—we have a situation where objective information conflicts with human intuition (that chewing a bar is bad for some reason, but chewing a stick is fine). The outdoor system has not flourished in numbers around the world. New farms in Asia or the Americas are rarely, if ever, outdoor systems. The outdoor system enjoys a positive consumer view when the weather is mild; however, if the weather is extreme, this system poses some challenges. Outdoor systems are perceived by consumers as good for animal welfare [36]. Yet, outdoor systems can pose threats to the well-being of animals, such as nutritional stress, inadequate water supply, parasitic diseases, wildlife-borne diseases, climatic extremes, lameness, predators, and a lower degree of human care and supervision [37]. A sustainability matrix comparing indoor and outdoor systems has been previously reported [38]. The negative issue with the outdoor system in modern times is about zoonotic diseases. Migratory and local birds (and other wildlife) carry diseases like influenza and Salmonella from one place to another. Calloway et al. [39] found that feed and water in an outdoor unit was continually seeded with Salmonella by birds, while contemporary pigs fed the same diets indoors had no signs of Salmonella infection. Multinational corporations could not take the liability risk of potential food safety concerns associated with the outdoor system and so (in the USA at least) the outdoor system is relegated to a local food, niche market opportunity for small pork producers. One author observed cattle in Inner Mongolia (a Provence of China) that were moved from pastures into buildings for the purpose of protecting ground water and soil from pollution. The local government and farmers did not view the loss of being outdoors as a negative. In one of the few surveys of this type, people in Spain believed animal welfare is better when they are outdoors expressing natural behaviors while Mexican consumers put more value on reducing pain [40].

## 4. Painful and Stressful Practices

### 4.1. Overview

One might wonder why a farmer would intentionally induce pain in a pig. These procedures are performed entirely for economic reasons. The goal is to eliminate boar taint, reduce piglet injury, and minimize tail biting. The simple solution would be to not perform painful procedures, but if economics drive their use, then finding ways to minimize the pain is desirable. Most painful procedures are conducted within a few days of age in piglets. These processing procedures consist of surgical castration, tail docking, ear tagging or notching, needle teeth clipping/grinding, injections with antibiotics, other medications, and transponders [41]. Pain management during processing is not common in the swine industry for any type of processing procedure (except in some countries).

### 4.2. Surgical Castration

Many studies have reported that surgical castration is painful [42,43]. Additionally, surgical castration can lead to acute physiological changes. These physiological changes include increased levels of adrenocorticotropic hormone (ACTH), cortisol, and lactate after castration, which are indicative of stress and tissue damage [44]. Surgical castration in piglets also induces behavioral changes. Strong vocal responses during castration have been widely reported in the literature [43], especially during castration without anaesthesia [45]. These high frequency calls are indicative of pain, mainly associated with pulling and severing of the spermatic cord [46]. Behaviors such as stiffness, prostration, and trembling are common for the first few hours after surgical castration but in the days following, piglets show other pain-like behaviors like scratching their rump and tail wagging [47].

The use of general anaesthesia can be induced by inhalation agents, such as halothane [48], isoflurane [49], carbon dioxide [50], and nitrous oxide [51], the latter two being less effective, due to an increase in distress in piglets.

The use of general anaesthetics and local anaesthetics may be limited by regulations and economics of the swine industry [41]. In addition, the use of anaesthetics is not approved in food animals by the FDA in the U.S. [41].

Alternatives to surgical castration include the use of chemical compounds to destroy testicular tissue [52], exogenous hormones to down-regulate the hypothalamic-pituitary-gonadal axis [53], immunocastration [54], marketing pigs at a younger age, and genetic selection against boar taint [39]. Yet, alternatives to surgical castration have not been permanently adopted in the U.S. and other countries and surgical castration remains a valid welfare concern [41].

### 4.3. Handling and Transport

Transport is an essential component in multi-site pork production used where farrowing, finishing, and processing occur in different locations [55]. Transport is a complex stressor for pigs made up of many factors, including condition of the pigs at the time of loading, handling, mixing of unfamiliar pigs, loading density/high stocking density, ambient temperature/fluctuating temperatures, withdrawal from feed and water, time in transit, motion, sudden speed changes, noise, and novelty, which can individually or in combination lead to injuries, and even death [56,57]. Stress associated with novelty such as handling and loading can cause physiological changes such as increased heart rate and immune changes [58]. The stress associated with transport can significantly impact animal welfare and lead to major meat quality defects, such as pale soft and exudative (PSE) and dark firm and dry (DFD) [59]. Transport losses include animals that are dead on arrival dead-on-arrival (DOA) or non-ambulatory (NA) upon arrival at the plant. These pigs are further categorized as non-ambulatory, injured which are unable/unwilling to walk (NAI), and pigs, which are non-ambulatory but not injured and are unwilling/unable to walk (NANI) [60].

Transport death can be painful and by no means an easy death, characterized by heart failure, and suffocation that may last from 10 min to 2 h [61]. Modern pigs have a lower tolerance for high temperatures during transport because they are bred for rapid growth, but their heart is small relative to their body size, and thus, acute stress during transport can lead to tachycardia and death due to heart failure [62]. Modern, fast-growing pigs might have pre-existing cardiac lesions and are possibly unable to respond to the cardiac workload required during sorting, loading, and transport [62].

Currently, there are few to no solutions to reduce all transport stress in pigs or other species.

### 4.4. Post-Mixing Aggression and Floor Space

Seaton Baxter (1984) wrote about animal needs in “confinement” or “intensive” systems/buildings in terms of pig needs for space and place [63]. This means, roughly, the quantity and quality of the environment we provide to our animals. Historically, enough space was provided to prevent performance suppression with little attention to the quality of the space or the effects of the developmental environment on pig social behaviors. Aggressive behavior provides an interesting case study of how our production system induces unwanted aggressive behaviors. We noticed that outdoor-born piglets do not fight as much as indoor-born piglets. In fact, indoor born weaned pigs fight when weaned and mixed, but outdoor born pigs did not fight after weaning, no matter if they are weaned into indoor or outdoor systems [64]. This finding is consistent with the idea that a lack of social development is the reason that pigs fight. It may be our production system that causes pig aggression or possibly the reduced amount of space compared to natural settings. In a more natural setting, fighting is a rare event. In modern systems, aggression is more common.

Aggression in pigs in a commercial setting can take place under two different conditions: (1) during mixing of unfamiliar pigs to establish a hierarchy and (2) competition for resources when they are scarce (such as when animals are overstocked). Pigs are usually mixed between production stages (such as from the farrowing barn to the nursery and from the nursery to the grower/finishing unit). It is common practice to also regroup pigs when sorting by size in the nursery or finishing barn. Lastly, mixing can occur during transport. Although many people attempt to load pigs of the same pen in the same compartment of the trailer, it is almost impossible to keep pen mates together. Consequently, mixing of unfamiliar pigs causes fighting, resulting in hurt/injured, fatigued, and stressed pigs [65].

Current strategies to reduce aggression consist of trying to avoid mixing of unfamiliar pigs. A study by Foister et al. [66] revealed that aggression within pens can be predicted. This research group found that unstable groups in newly mixed pens of pigs is likely to lead to prolonged chronic aggression and elevated injury rates, whereas pens with large cliques (about 47% of the pen members) are likely to have significantly fewer injuries in stable groups. Post-mixing aggression can lead to harmful and costly behavior, which impacts pig welfare and farm efficiency [66].

Market weight has increased by 5.8 kg every 10 years for the past four decades, driven by the dilution of fixed cost over more weight per pig, improved genetics, and nutrition that results in more efficient pigs [67]. Although pig market weights vary depending on regions and cultural backgrounds, as of the beginning of 2017, in the U.S., the average market weight was reported to be 129 kg, but varied between 124 to 130 kg in 2016 [68]. The increase in pig weights can lead to increased stocking densities towards the end of the finishing phase, increasing feeder and water competition, thus causing aggression among pen mates that can lead to an increase in injuries, lameness, among other problems. Thus, further research and production system remedies to this issue are warranted.

## 5. Conclusions and Further Studies

The modern pig is quite different in anatomy and behavior than its wild ancestor. Domestication and selective breeding have resulted in a modern pig that is lean, fast-growing, efficient, with a very large litter size compared to the European Wild Boar. The characteristics of the modern pig can make it more susceptible to certain conditions, such as during handling and transport, as these pigs have a lower tolerance for high temperatures.

While behaviors may have changed through domestication in level through selective breeding, few behaviors have been eliminated or added. Compared to wild pigs, the modern, domestic pig retained its large appetite, its motivation to root and dig, and social behaviors. Modern production systems and a lack of skilled labour contribute to welfare challenges on commercial farms. The degree of on-farm animal welfare oversight varies tremendously around the world. Often, but not always, science is used to set animal welfare rules. However, science sometimes conflicts with cultural values. Producers in some less-developed countries are being asked to follow animal welfare rules based on an ethical position held by others. This ethical construct should be examined. Some authors even argue we should use our economic strength to make countries with developing economies comply with animal welfare rules [13]. We believe that we should use science to determine animal welfare needs of farm pigs but with a healthy respect for cultural differences in the ethical mandates for improved animal care.

## Data Availability

Not applicable.
